# Transplanting cells from old but not young donors causes physical dysfunction in older recipients

**DOI:** 10.1111/acel.13106

**Published:** 2020-01-23

**Authors:** Binsheng Wang, Zukai Liu, Vicky P. Chen, Lichao Wang, Christina L. Inman, Yueying Zhou, Chun Guo, Tamar Tchkonia, David W. Rowe, George A. Kuchel, Paul Robson, James L. Kirkland, Ming Xu

**Affiliations:** ^1^ UConn Center on Aging UConn Health Farmington Connecticut; ^2^ Department of Genetics and Genome Sciences UConn Health Farmington Connecticut; ^3^ Biomedical Science Graduate Program UConn Health Farmington Connecticut; ^4^ Department of Molecular Pharmacology and Experimental Therapeutics Mayo Clinic Rochester Minnesota; ^5^ Robert and Arlene Kogod Center on Aging Mayo Clinic Rochester Minnesota; ^6^ Xiangya Stomatological Hospital Central South University Changsha China; ^7^ Center for Regenerative Medicine and Skeletal Development UConn Health UConn Health Farmington Connecticut; ^8^ The Jackson Laboratory for Genomic Medicine Farmington Connecticut

**Keywords:** aging, cellular senescence, frailty, regenerative medicine

## Abstract

Adipose‐derived mesenchymal stem cell (ADSC)‐based regenerative therapies have shown potential for use in many chronic diseases. Aging diminishes stem cell regenerative potential, yet it is unknown whether stem cells from aged donors cause adverse effects in recipients. ADSCs can be obtained using minimally invasive approaches and possess low immunogenicity. Nevertheless, we found that transplanting ADSCs from old donors, but not those from young donors, induces physical dysfunction in older recipient mice. Using single‐cell transcriptomic analysis, we identified a naturally occurring senescent cell‐like population in ADSCs primarily from old donors that resembles in vitro‐generated senescent cells with regard to a number of key pathways. Our study reveals a previously unrecognized health concern due to ADSCs from old donors and lays the foundation for a new avenue of research to devise interventions to reduce harmful effects of ADSCs from old donors.

## Graphical abstract

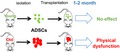

Adipose‐derived mesenchymal stem cells (ADSCs) or “preadipocytes” have been increasingly suggested for use in regenerative medicine as a treatment for a wide range of diseases due to their multipotency and accessibility. Older adults represent most likely recipients of ADSC therapies given the high burden of diseases in this population. Since autologous ADSCs are preferred in the clinic, it is essential to understand age‐related changes influencing these cells. Emerging evidence suggests that ADSCs from aged donors have reduced regenerative potential, leading to diminished therapeutic efficacy (Khong et al., 2019; Liu et al., 2017; Ye et al., 2016). However, it is unknown whether transplanting ADSCs from aged donors might cause unexpected or even harmful effects in recipients. This is especially important for older adults, since they tend to be more vulnerable and less resilient to such stresses.

To examine this, we isolated ADSCs from 12 young (6–7 months, referred to as young ADSCs) and 12 old (28–31 months, referred to as old ADSCs) C57BL/6 male mice. We transplanted 1 x 10^6^ ADSCs from young or old donors i.p*.* into syngeneic 20‐month‐old C57BL/6 male mice (Figure 1a). Four to six weeks after transplantation, we tested maximal walking speed (RotaRod), grip strength, physical endurance (hanging test, treadmill), daily activity, food intake, and body weight change to assess overall physical function in recipients, based on criteria used in clinical practice (Fried et al., 2001). ADSCs from old donors significantly impaired walking speed, grip strength, endurance, and daily activity of older recipient mice after transplantation, compared with mice transplanted with the same number of ADSCs from young donors (Figure 1b‐e). No statistically significant differences were observed in terms of treadmill, body weight change, food intake, or remaining lifespan (Figure S1a‐d), which might be due to an insufficient number of transplanted old ADSCs to cause detectible changes in these tests. Overall, these findings suggest that ADSCs from old donors can induce physical frailty, which is highly associated with morbidity and loss of independence(Ensrud et al., 2009). Thus, regenerative approaches entailing transplantation of ADSCs from aged donors might generate previously unrecognized risks.

**Figure 1:**
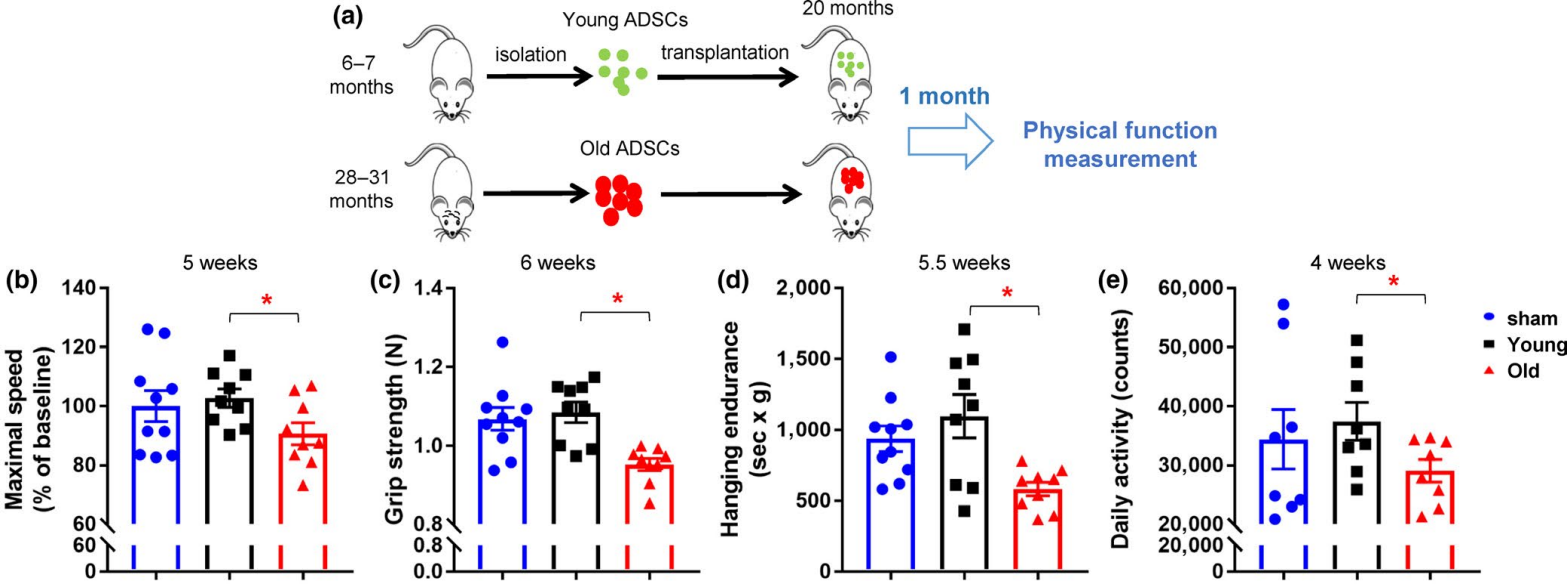
Adipose‐derived mesenchymal stem cell (ADSCs) from old donors impair physical function. (a) Experimental design. (b‐e) Quantification of maximal walking speed (relative to baseline) (b), grip strength (c), hanging endurance (d), daily activity (e) of 21‐month‐old male C57BL/6 mice 4–6 weeks after being injected with 1 × 10^6^ ADSCs from old or young donors or no (sham) ADSCs. For b, c, d, *n* = 10 for sham, *n* = 9 for Young, *n* = 9 for old. For e, *n* = 8 for all groups. Results are mean ± *SEM*. *, *p* < .05; Student's *t* test

In a previous study, we demonstrated that transplanting a small number of in vitro‐generated senescent ADSCs (stable proliferative arrest caused by various stresses (Campisi & d'Adda di Fagagna, 2007) induces physical dysfunction in mice (Xu et al., 2018). To define a potential naturally occurring senescent cell‐like population, we conducted single‐cell transcriptome (SCT) analysis. We obtained high‐quality single‐cell transcriptomes (5,565 genes and 33,811 polyadenylated RNA transcripts detected per cell on average) from 3,604 and 1,876 ADSCs from young and old donors, respectively. Cdkn1a (*p21*) and Cdkn2a (*p16*) are two markers frequently associated with cellular senescence (Campisi & d'Adda di Fagagna, 2007), both of which were highly expressed in cells isolated from the old donors (Figure 2a). We manually set a cutoff of *p21* expression level, which is a higher expression level than in 97% of cells from young donors. We considered cells to be *p21* ^high^ if their *p21* levels were higher than this cutoff. We found that 8.9% of cells from old donors were *p21* ^high^ compared with 3% of cells in young donors (Figure 2a), both of which are similar to the percentages of senescent cells observed in ADSCs isolated from young and old human donors (Xu et al., 2015). We used *p21* rather than *p16* because the difference of *p21* levels between young and old cells was more significant than *p16* (Figure 2a,c) and *p16* level positively correlates with *p21* in *p21* ^high^ cells (*p* < .001). In addition, we found that cells from old donors proliferated at a slower rate and contained more SA‐βgal^+^ and *p21* ^high^ cells compared with the cells from young donors (Figure S2), which is consistent with an increased abundance of senescent cells among the cells isolated from the old donors. Notably, we documented the transcriptomic signatures of naturally occurring *p21* ^high^ senescent‐like cells for the first time. By comparing all the distinctly expressed genes within this population using Ingenuity Pathway Analysis (IPA), we observed altered upstream regulators and pathways (Figure 2b) in these naturally occurring *p21* ^high^ cells that are similar to those of in vitro‐generated “artificial” senescent cells, including Senescent Cell Anti‐Apoptotic Pathways (SCAPs; increased cell survival and decreased apoptosis) (Zhu et al., 2015), NF‐κB (Chien et al., 2011), IL6/JAK (Xu et al., 2015), mTOR (Herranz et al., 2015; Laberge et al., 2015), FOXO (Baar et al., 2017), and HMGB1 (Davalos et al., 2013) pathways. These findings suggest that *p21* ^high^ cells might contribute to the physical dysfunction and validate the physiological relevance of the in vitro‐generated senescent cells that have been extensively used to study the biology of senescence and for drug screening. To gain more insight into deleterious effects of aging on the ADSCs, we compared transcriptomes of ADSCs isolated from young versus old donors using edgeR (Robinson, McCarthy, & Smyth, 2010). Among 15,565 detected genes, 3,787 were up‐regulated and 948 were down‐regulated (–log_10_ *P* value > 4) in the cells from old donors (Figure 2c and Supporting dataset). Several senescence‐associated secretory phenotype (SASP)‐related genes were up‐regulated in cells from old donors (Figure 2c). For validation, we measured secreted protein levels of these genes in conditioned media from young versus old ADSCs by multiplex protein analysis (Xu et al., 2015) . In most cases, the mRNA differences were consistent with differences in secreted proteins (Figure 2d). Using IPA, we detected a number of pathways that were distinctly expressed in ADSCs isolated from young versus old donors (Figure 2e), affording an opportunity to develop interventions that could reverse aged ADSCs into a more “youthful” state, potentially reducing harmful effects and improving regenerative efficacy. One limitation of our study is that we used allogenic transplantation (although the donors and the recipients were syngeneic), which might not fully represent autologous transplantation used in clinic. In future studies, more focused, in‐depth mechanistic insights into how ADSCs from old donors exert their adverse effects need to be obtained. Potential mechanisms include cellular senescence, inflammaging, and resulting sarcopenia. Also, SCT analysis using other senescence‐related markers such as p16, Il6, and Cxcl1 would be helpful for understanding the biology of senescence.

**Figure 2:**
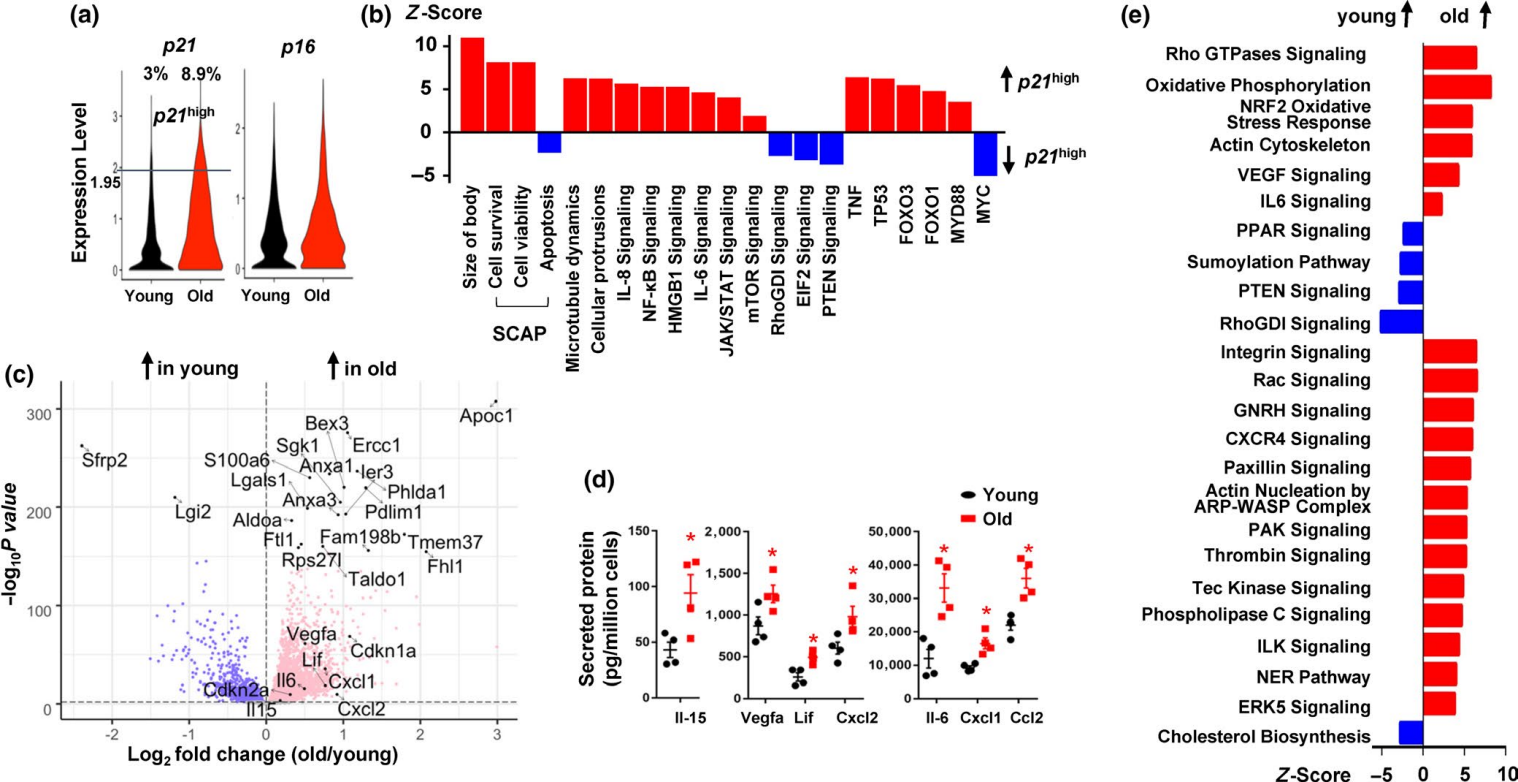
Single‐cell transcriptome analysis. (a) Violin plots for *p21* and *p16* expression levels in young and old cell populations. (b) IPA analysis of *p21* ^high^ cells. Positive Z‐score indicates up‐regulation in *p21* ^high^ cells. (c) Volcano plot of differentially expressed genes between cells from young and old donors. The top 20 most significantly altered genes, and several selected genes are highlighted. –log_10_ P value and log_2_(fold change) values are shown. (d) Secreted cytokine levels in conditioned media from ADSCs isolated from young and old donors. *n* = 4. Results are mean ± *SEM*. *, *p* < .05; Student's *t* test. (e) IPA analysis of canonical pathways enriched in adipose‐derived mesenchymal stem cell (ADSCs) from old versus young donors

In summary, we demonstrate that old ADSCs can impair physical function in older recipients, indicating a potential health concern regarding transplanting aged ADSCs. By SCT, we identified a naturally occurring *p21* ^high^ senescent‐like cell population. Currently, there is limited knowledge about in vivo senescent cells due to the difficulty in isolating them because of their low numbers and lack of ideal markers. To our knowledge, we obtained the transcriptomic signatures of in vivo* p21* ^high^ senescent‐like cells for the first time, which could assist in understanding the biology of senescence and drug development. In addition, we uncovered many signaling pathways that vary between ADSCs isolated from young versus old donors. Our study potentially begins new avenues of research to discover whether pharmacological interventions, such as senolytic drugs (Tchkonia & Kirkland, 2018) or anti‐inflammatory drugs, can prevent or reverse dysfunction caused by transplanting ADSCs or even organs from old donors (Lau, Kennedy, Kirkland, & Tullius, 2019) and improve clinical outcomes of transplantation for older patients.

## CONFLICT OF INTERESTS

M.X., T.T., and J.L.K, have a financial interest related to this research. Patents on senolytic drugs (including PCT/US2016/041646, filed at the US Patent Office) are held by Mayo Clinic.

## AUTHOR CONTRIBUTIONS

M.X. conceived and designed the study. M.X., B.W., V.P.C., C.L.I., C.G., and T.T. performed the mouse studies. B.W., Z.L., M.X., and P.R. contributed to SCT analysis. B.W., L.W., Y.Z., and D.W.R. contributed to the cellular experiments. G.A.K. and J.L.K. contributed to the manuscript preparation. M.X. wrote the manuscript with input from all coauthors. M.X. and J.L.K. oversaw all experimental design, data analysis, and manuscript preparation.

## Data Availability

Raw data are openly available in “figshare” at https://doi.org/10.6084/m9.figshare.11295950.v1
