# Supplementary figures and images for: Transplanting cells from old but not young donors causes physical dysfunction in older recipients
Source: Aging Cell. 2020 Jan 23;19(3):e13106. doi: 10.1111/acel.13106 (PMC7059132; doi:10.1111/acel.13106)

Figure S1

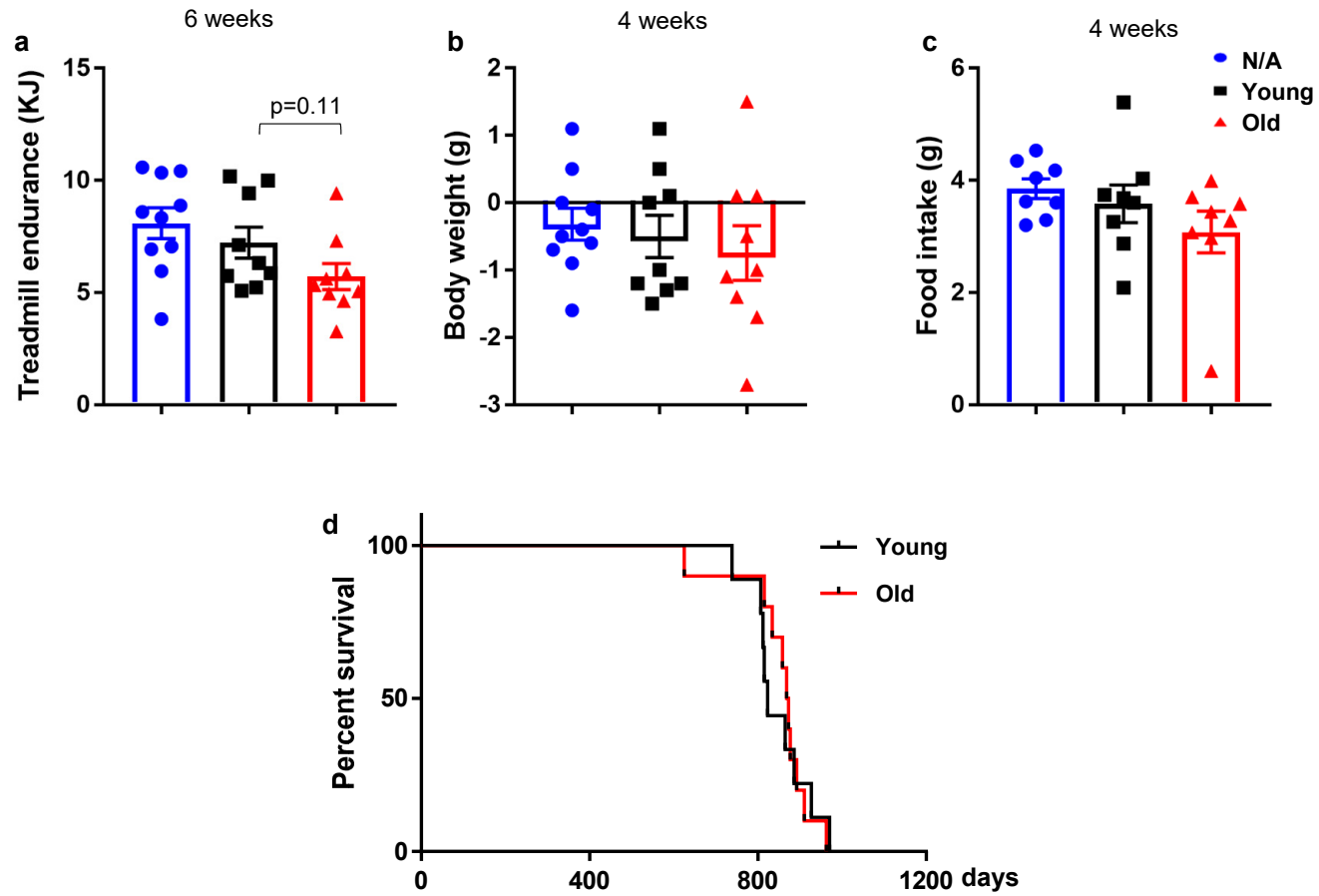

Figure S2

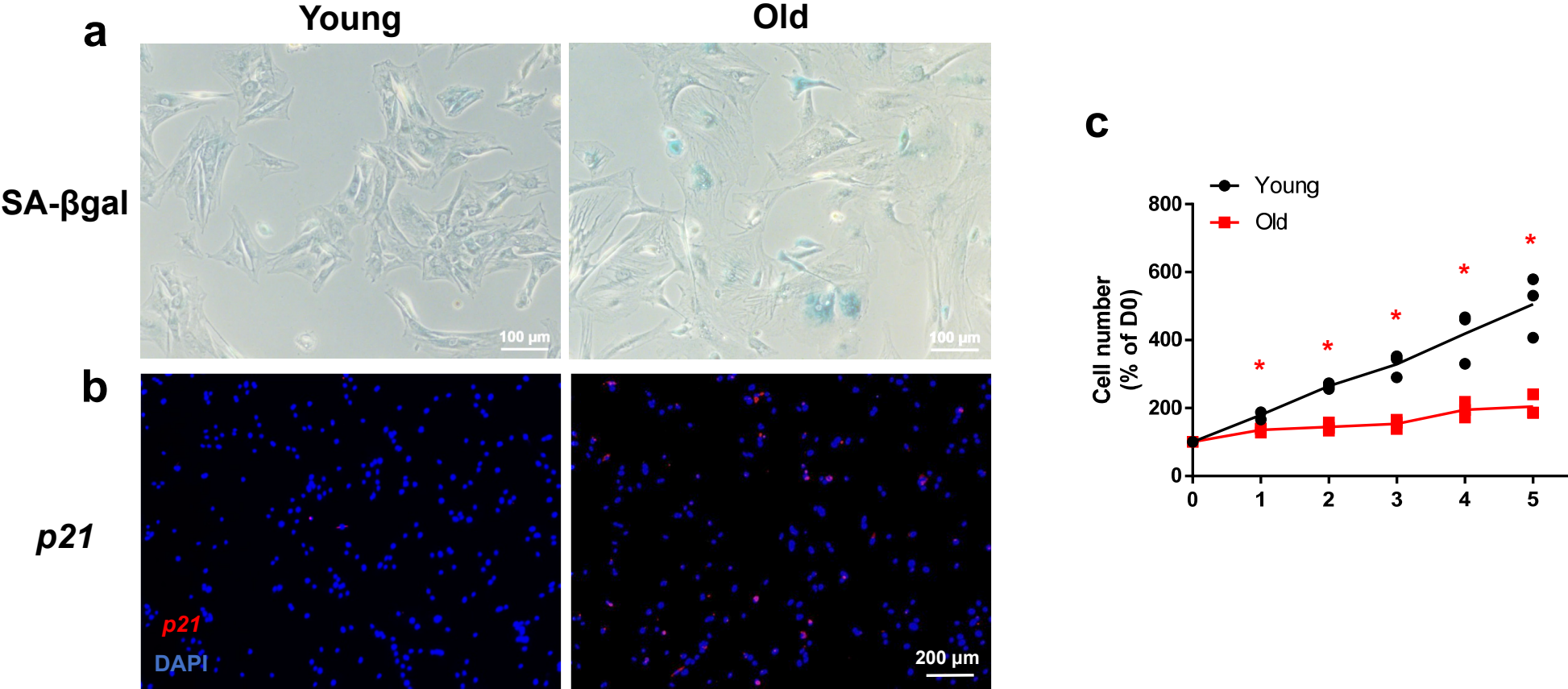

Figure S3

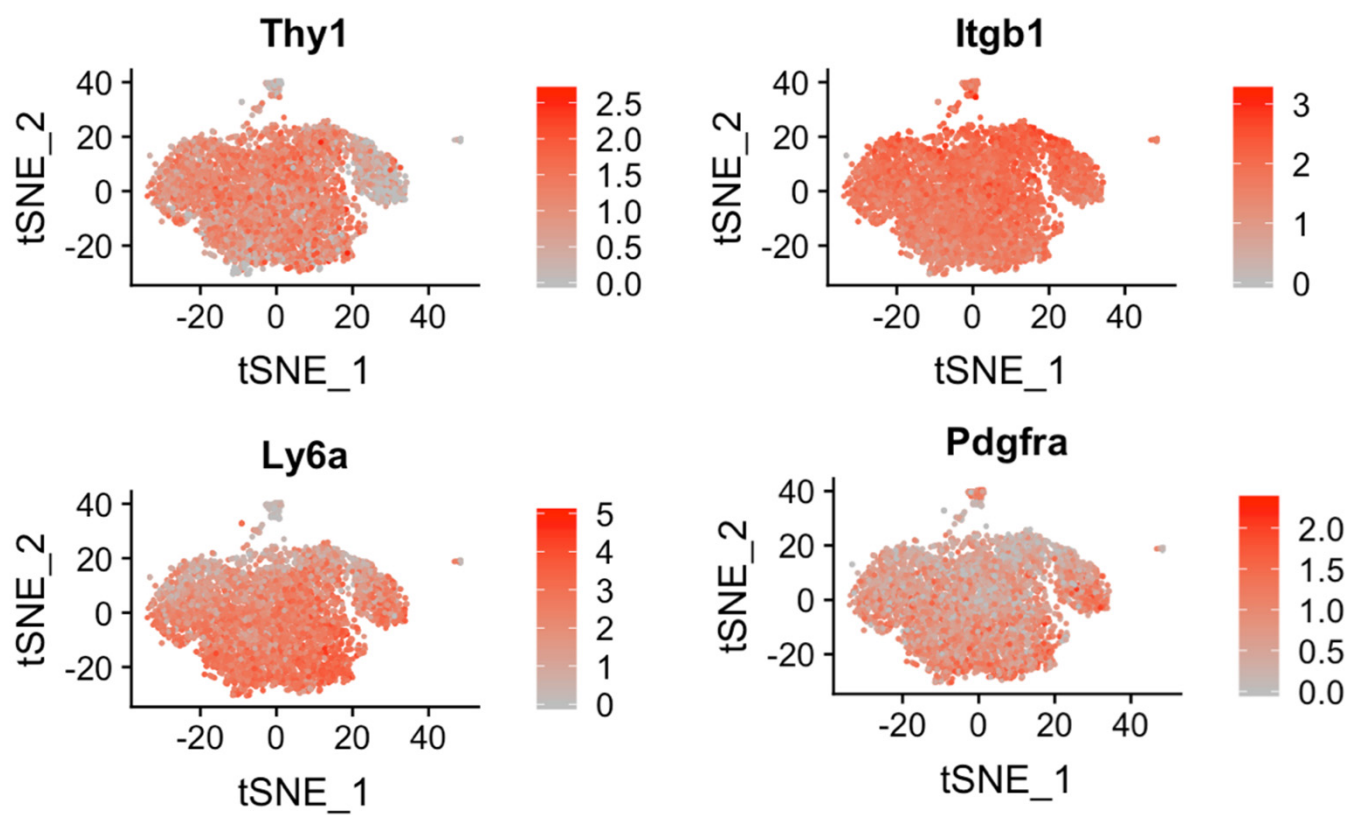

Figure S4

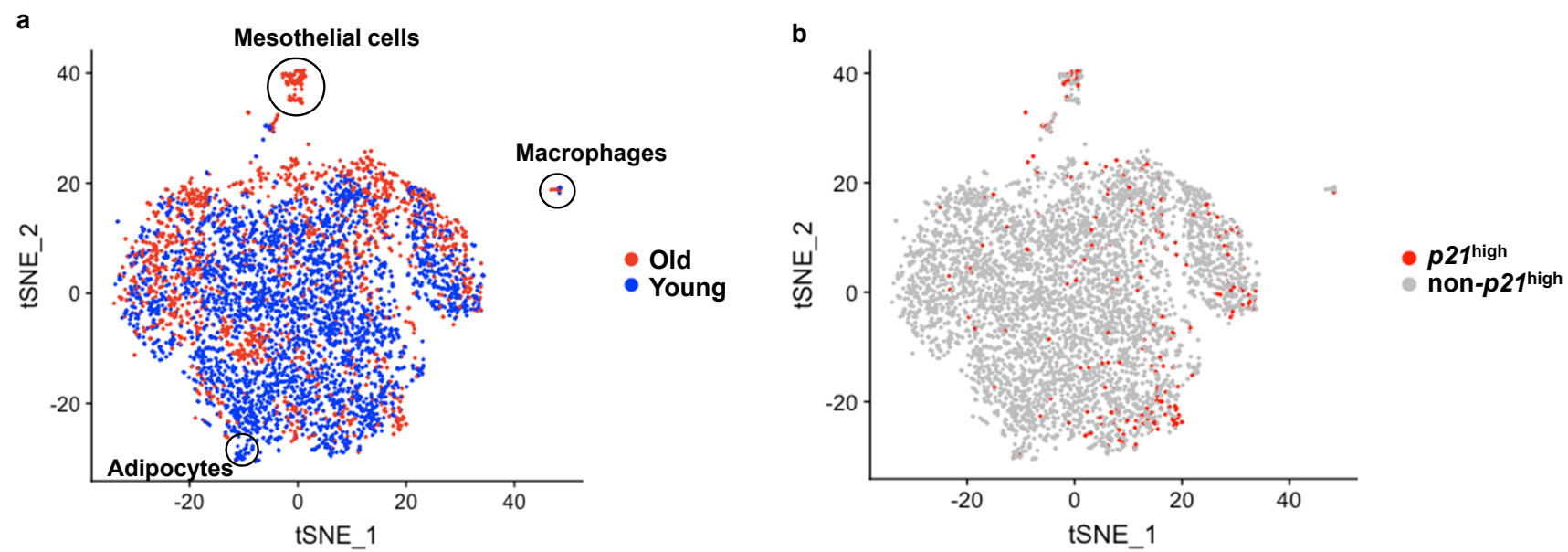

Supplement: Supplementary file 1 [file ACEL-19-e13106-s001.pdf]
