# Supplementary material for: Transplanting cells from old but not young donors causes physical dysfunction in older recipients
Source: Aging Cell. 2020 Jan 23;19(3):e13106. doi: 10.1111/acel.13106 (PMC7059132; doi:10.1111/acel.13106)
Supplement: Supplementary file 2 [file ACEL-19-e13106-s002.docx]

**Supplementary information**

**Materials and Methods**

**Mouse models**

All animal experiments were performed according to protocols approved by the Institutional Animal Care and Use Committee (IACUC) at Mayo Clinic. Wild-type C57BL/6 mice were obtained from the National Institute on Aging (NIA) and maintained in a pathogen-free facility at 23–24 °C under a 12-hr light, 12-hr dark regimen with free access to a NCD (standard mouse diet with 20% protein, 5% fat (13.2% fat by calories), and 6% fiber; Lab Diet 5053, St. Louis, MO) and water. All physical function measurements were performed as previously published[^1^](#_ENREF_1)^,^[^2^](#_ENREF_2). For each test, all the groups were assessed at the same time point to get comparable results.

**ADSC isolation and characterization**

Mouse ADSCs were isolated from all fat depots as described previously[^2^](#_ENREF_2)^,^[^3^](#_ENREF_3). None of the donor mice had evident tumors. Briefly, after euthanasia, fat tissue was removed under sterile conditions from mice, minced, and then digested in Hank's balanced salt solution (HBSS) containing 1 mg/ml collagenase in a 37°C shaking water bath for 1 hour until fragments were no longer visible and the digest had a milky appearance. Digests were filtered through a 100-μ m nylon mesh and centrifuged at 1200rpm for 10 min. Supernatant including adipocytes was carefully aspirated, and the cell pellets were washed with PBS once and then plated in α-MEM containing 10% calf serum and antibiotics (All from Thermo Fisher Scientific, Waltham, MA). After 12 h, adherent cells were washed, trypsinized, and replated in order to reduce potential endothelial cell and macrophage contamination. ADSCs isolated using this protocol have been well characterized including adipogenic capacity, osteogenic differentiation, and chondrogenic differentiation in previous studies[^4^](#_ENREF_4)^,^[^5^](#_ENREF_5). Using SCT, we found that the vast majority of cells were contained within neighboring clusters all sharing classical ADSC/mesenchymal markers[^6^](#_ENREF_6), including CD90/Thy1, CD29/Itgb1, Sca1/Ly6a, and Pdgfra (Fig.S3). Smaller outlying clusters were identified as non-ADSCs (Fig.S4), including macrophages, mesothelial cells, and a few endothelial cells. Their presence likely represents differentiation or carry-over from the ADSC isolation procedure. These non-ADSCs were excluded from analysis to avoid any potentially confounding effects.

**Multiplex protein analyses**

ADSCs were cultured in conditioned media (CM) for 24 hrs. Each replicate represents a mixture of cells from 3 donors. Pro-inflammatory cytokine and chemokine protein levels in CM were measured using Luminex xMAP technology. The multiplexing analysis was performed using the Luminex 100 system (Luminex, Austin, TX) by Eve Technologies Corp. (Calgary, Alberta, Canada).

**Cell transplantation**

Cells were passaged for one round in physiological (3%) oxygen before transplantation. 1x10^6^ ADSCs isolated from young (6-7 months) or old (28-31 months) mice were transplanted into syngeneic 20-month-old C57BL/6 male mice by *i.p.* injection as previously published[^1^](#_ENREF_1). The total numbers of recipients received ADSCs isolated from young or old donors were 9 and 9, respectively. Another 10 recipients only received one injection of PBS as the sham group. Transplanted ADSCs mainly localized to visceral fat and survived *in vivo* for at least 40 days based on our previous study[^1^](#_ENREF_1).

**Single cell transcriptome analysis**

Due to the heterogeneity of ADSCs, we reasoned that SCT could yield more refined insight into age-related changes in ADSCs than bulk RNA sequencing. High quality single cell transcriptomes were obtained from 3,604 young and 1,876 aged ADSCs using a droplet-based 10X genomics platform[^7^](#_ENREF_7). These data were generated in a single channel using pooled samples from young and old biological replicates using cell hashing[^8^](#_ENREF_8) to ensure differences between young and old wee a result of biological differences rather than technical artifacts. These deep single cell data - 5,565 genes and 33,811 polyadenylated RNA transcripts detected *per* cell on average – were then analyzed to identify distinct populations of cells, assisted by the Seurat package[^9^](#_ENREF_9). Differentially expressed genes among different ADSCs were analyzed by edgeR^[10](#_ENREF_10)^, and pathway and upstream regulator analyses were done using Ingenuity Pathway Analysis (IPA) software.

**Cell proliferation assay**

ADSCs were seeded at 2,000 cells *per* well into 96-well plates overnight. The number of viable cells was detected using a CellTiter-Glo Luminescent Cell Viability Assay kit (G7572, Promega, Madison, WI) according to the manufacturer’s instructions.

**Immunostaining and senescence-associated β-galactosidase (SA-βgal)** **assay**

ADSCs were seeded into 12-well plate overnight and fixed in 4% paraformaldehyde for 15 minutes. Cells were then permeabilized with 0.3% Triton X-100 for 10 minutes, followed by blocking with 1% BSA for 30 minutes. After being exposed to anti-p21 antibody (sc-6246, Santa Cruz Biotechnology, Dallas, TX) overnight at 4°C, cells were incubated with Alexa Fluor 647-conjugated secondary antibody (ab150103, Abcam, Cambridge, MA) for 1 hour. Cells were subsequently counterstained with Hoechst 33342 (H3570, Invitrogen, Carlsbad, CA) and imaged on a fluorescence microscope (Zeiss, Jena, Germany). The SA-βgal assay was performed as described previously^2^.

**Supplementary References**

1. Xu, M.*, et al.* Senolytics improve physical function and increase lifespan in old age. *Nature medicine* **24**, 1246-1256 (2018).

2. Xu, M.*, et al.* JAK inhibition alleviates the cellular senescence-associated secretory phenotype and frailty in old age. *Proceedings of the National Academy of Sciences of the United States of America* **112**, E6301-6310 (2015).

3. Xu, M.*, et al.* Targeting senescent cells enhances adipogenesis and metabolic function in old age. *Elife* **4**, e12997 (2015).

4. Liu, M.*, et al.* Adipose-Derived Mesenchymal Stem Cells from the Elderly Exhibit Decreased Migration and Differentiation Abilities with Senescent Properties. *Cell Transplant* **26**, 1505-1519 (2017).

5. Ye, X.*, et al.* Age-Related Changes in the Regenerative Potential of Adipose-Derived Stem Cells Isolated from the Prominent Fat Pads in Human Lower Eyelids. *PLoS One* **11**, e0166590 (2016).

6. Merrick, D.*, et al.* Identification of a mesenchymal progenitor cell hierarchy in adipose tissue. *Science* **364**(2019).

7. Zheng, G.X.*, et al.* Massively parallel digital transcriptional profiling of single cells. *Nature communications* **8**, 14049 (2017).

8. Stoeckius, M.*, et al.* Cell Hashing with barcoded antibodies enables multiplexing and doublet detection for single cell genomics. *Genome Biol* **19**, 224 (2018).

9. Stuart, T.*, et al.* Comprehensive Integration of Single-Cell Data. *Cell* **177**, 1888-1902 e1821 (2019).

10. Robinson, M.D., McCarthy, D.J. & Smyth, G.K. edgeR: a Bioconductor package for differential expression analysis of digital gene expression data. *Bioinformatics* **26**, 139-140 (2010).

**Fig.S1** **ADSCs from old donors impair physical function.** Treadmill endurance (*a*), body weight change from baseline (*b*), and food intake (*c*) in 21-month-old male C57BL/6 mice 4-6 weeks after being injected with 1 × 10^6^ ADSCs from old or young donors or no (N/A) ADSCs. For a and b, n= 10 for N/A, n=9 for Young, and n=9 for Old. (*d*) Survival curves of 20 month old recipient mice (n=9 for Young, n=10 for Old).

**Fig.S2** **ADSCs from old donors contain more senescent cells.** SA-βgal staining *(a)*, p21 immunostaining *(b)*, and cell proliferation rate *(c)* are shown in cells isolated from young or old donors.

**Fig.S3 ADSCs used in this study express key ADSC markers.** Expression patterns of selected cell markers on a t-distributed stochastic neighbor embedding (tSNE) map.

**Fig.S4 ADSCs from old donors contain more *p21*^high^ cells.** Unsupervised clustering of single cell sequencing data on a tSNE map showing ADSCs from old *vs.* young donors (*a*) and *p21*^high^ *vs*. non-*p21*^high^ ADSCs (*b*).

**Supporting dataset.** Gene list for comparing transcriptomes of young vs. aged ADSCs. Log (fold change (FC)) and p-value were shown for each gene.
